# Inflammatory Nasal Polyp With Benign Lymphangioendothelioma/Acquired Progressive Lymphangioma in a Young Man

**DOI:** 10.7759/cureus.68079

**Published:** 2024-08-29

**Authors:** Kenji Yorita, Katsushi Miyazaki, Masanori Hisaoka, Kimiko Nakatani

**Affiliations:** 1 Diagnostic Pathology, Japanese Red Cross Kochi Hospital, Kochi, JPN; 2 Otolaryngology, Japanese Red Cross Kochi Hospital, Kochi, JPN; 3 Pathology and Oncology, School of Medicine, University of Occupational and Environmental Health, Kitakyushu, JPN; 4 Radiology, Japanese Red Cross Kochi Hospital, Kochi, JPN

**Keywords:** pathology, nasal septum, acquired progressive lymphangioma, benign lymphangioendothelioma, nasal polyp

## Abstract

A 17-year-old Japanese male presented with a nasal polyp and neck lymphadenopathy. He was referred to our hospital for diagnosis and treatment of neck lymphadenopathy. Grossly, a whitish pedunculated nasal polyp was observed on the right side of the nasal septum. Computed tomography confirmed the presence of a nasal polyp and two nodular lesions in the right maxillary sinus. All three lesions showed no contrast enhancement. Clinical examination revealed neck lymphadenopathy, suggestive of Kikuchi disease. The nasal polyp was resected, and pathological examination revealed an inflammatory nasal polyp with benign lymphangioendothelioma, also known as acquired progressive lymphangioma. The right maxillary sinus nodules were followed up. The nasal occurrence of benign lymphangioendothelioma is markedly unusual, as the lesion typically occurs in the skin. To the best of our knowledge, this is the first reported case of nasal benign lymphangioendothelioma in our review of the PubMed database.

## Introduction

Nasal polyps are benign tumors that occur in the nasal cavity or sinuses. They are usually caused by chronic inflammation and are more common in individuals with allergies or asthma. However, in rare cases, nasal polyps can be caused by other conditions, such as infections or tumors.

Benign lymphangioendothelioma (BL), also known as acquired progressive lymphangioma (APL), is a rare condition histologically characterized by benign lymphatic vascular proliferation with infiltrative lymphatic channels dissecting the collagen [1]. Although BL/APL histologically shows infiltrative growth, it does not exhibit malignant behavior such as metastasis or invasion into adjacent tissues. Although a case study showed that BL/APC can show rapid progression, and multisite involvement requires exploration of nonsurgical options for management [2], the benign nature and gradual enlargement in other cases suggest that the term "aggressive" should be considered in the context of its histological growth pattern rather than its clinical outcome [3].

A recent literature review of BL/APL from 1963 to 2022 confirmed that 83 patients had BL/APL [3]. The skin is a typical site of BL/APL. This case report describes a 17-year-old Japanese man with a nasal polyp and BL/APL, revealing an unusual site for the lesion.

## Case presentation

A 17-year-old Japanese male with swollen lymph nodes on the posterior side of the right neck was referred to our hospital. The patient took no medication and had no history of food or medication allergies. The patient reported a medical history of childhood asthma. He had a coronavirus infection six months before presenting to the hospital.

He had experienced recurrent fever and a painful right posterior neck lesion for three months before presentation. When he visited his local doctor, he was diagnosed with lymphadenitis and received antibiotics as a diagnostic treatment; however, the lesion remained unchanged. At our hospital, a physical examination revealed no palpable enlarged lymph nodes; however, ultrasonography confirmed multiple swollen lymph nodes in the right posterior neck area, with a maximum lymph node size of 16 mm. Blood test results indicated a pre-existing infection pattern for Epstein-Barr virus and cytomegalovirus, while tests for *Toxoplasma *and antinuclear antibodies were negative. The white blood cell count was within the standard range. Together with the lack of response to antibiotics, bacterial lymphadenitis was unlikely. Given the presence of neck lymphadenopathy, the possibility of Kikuchi's disease was raised, and the patient was placed under observation.

**Figure 1:**
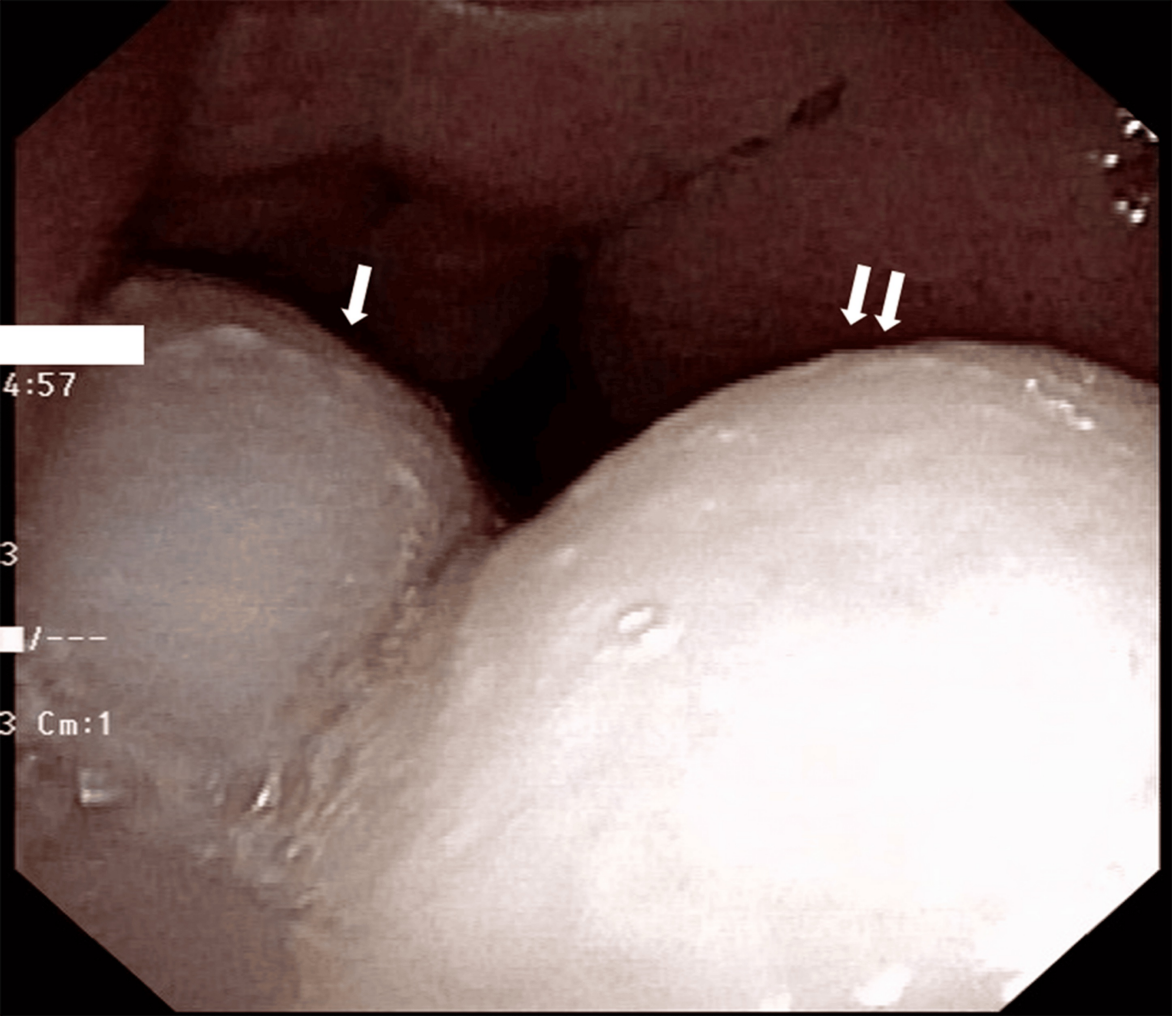
Endoscopic findings of the inflammatory nasal polyp with benign lymphangioendothelioma/acquired progressive lymphangioma. A whitish polyp with a two-humped surface indicated by one and double arrows is observed.

In addition to neck lymphadenopathy, a nodular lesion was observed in the left nostril. Endoscopy confirmed a whitish lesion in the left posterior nostril (Figure 1).

**Figure 2:**
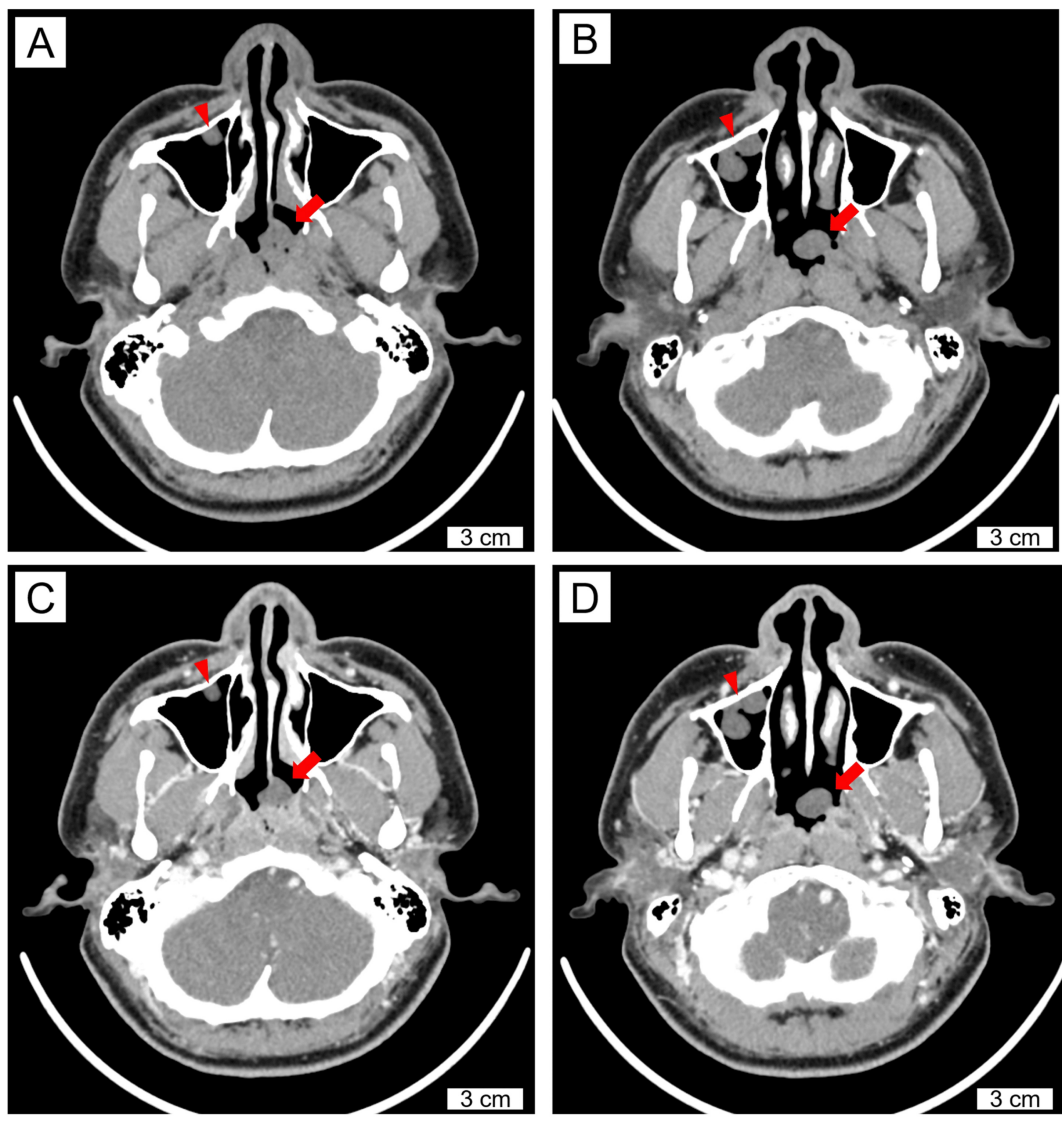
Computed tomographic images of the inflammatory nasal polyp with benign lymphangioendothelioma/acquired progressive lymphangioma. Computed tomography images (A, B: non-contrast-enhanced; C, D: contrast-enhanced; A corresponds to C; B corresponds to D) show a non-enhanced low-density lesion in the posterior nasal cavity (arrow) and two non-enhanced polypoid lesions in the right maxillary sinus (arrowhead). The nasal polyp attaches to the nasal septum in A and C. Scale bars are shown in the figures.

The polyp showed an unusual gross appearance of inflammatory nasal polyps, and respiratory epithelial adenomatoid hamartoma and juvenile angiofibroma were considered in the differential diagnosis. Contrast-enhanced computed tomography of the head revealed a non-enhanced low-density lesion in the nasal polyp and two non-enhanced nodular lesions in the right maxillary sinus (Figure 2).

Respiratory epithelial adenomatoid hamartoma might be suggested, and a nasal polyp was resected for pathological diagnosis. During the surgery, the nasal polyp was pedunculated and connected to the posterior portion of the nasal septum.

**Figure 3:**
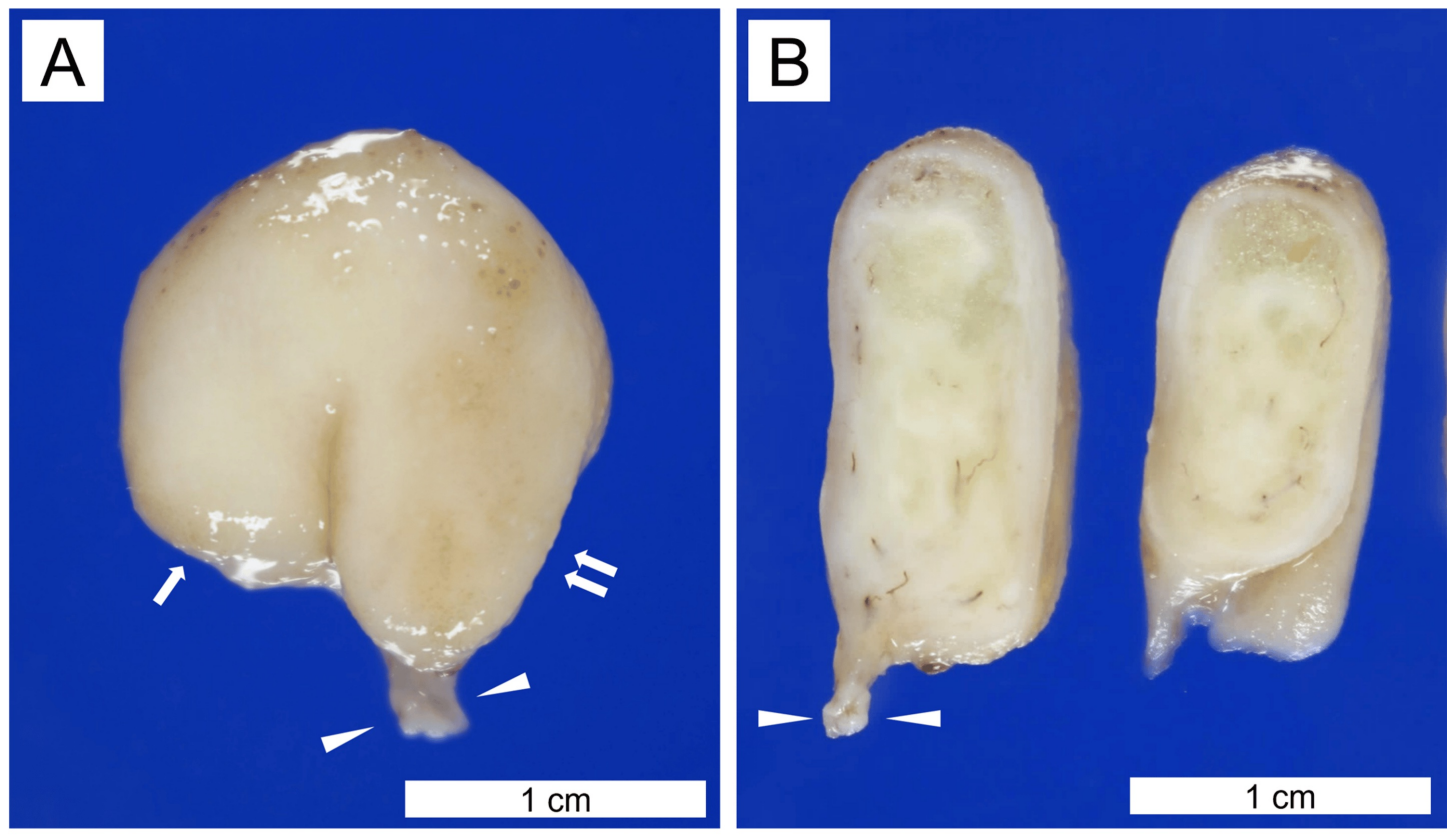
Gross findings of the inflammatory nasal polyp with benign lymphangioendothelioma/acquired progressive lymphangioma. Formalin-fixed resected tissue (A, external image; B, cut surfaces) shows a pedunculated polyp with a narrow stalk (indicated by arrowheads) and white irregularly edematous cut surfaces. The two-humped surface is indicated by one and two arrows in A. Scale bars are shown in the figures.

Grossly, the nasal polyp, measuring up to 2 cm in size, displayed white, edematous cut surfaces (Figure 3).

**Figure 4:**
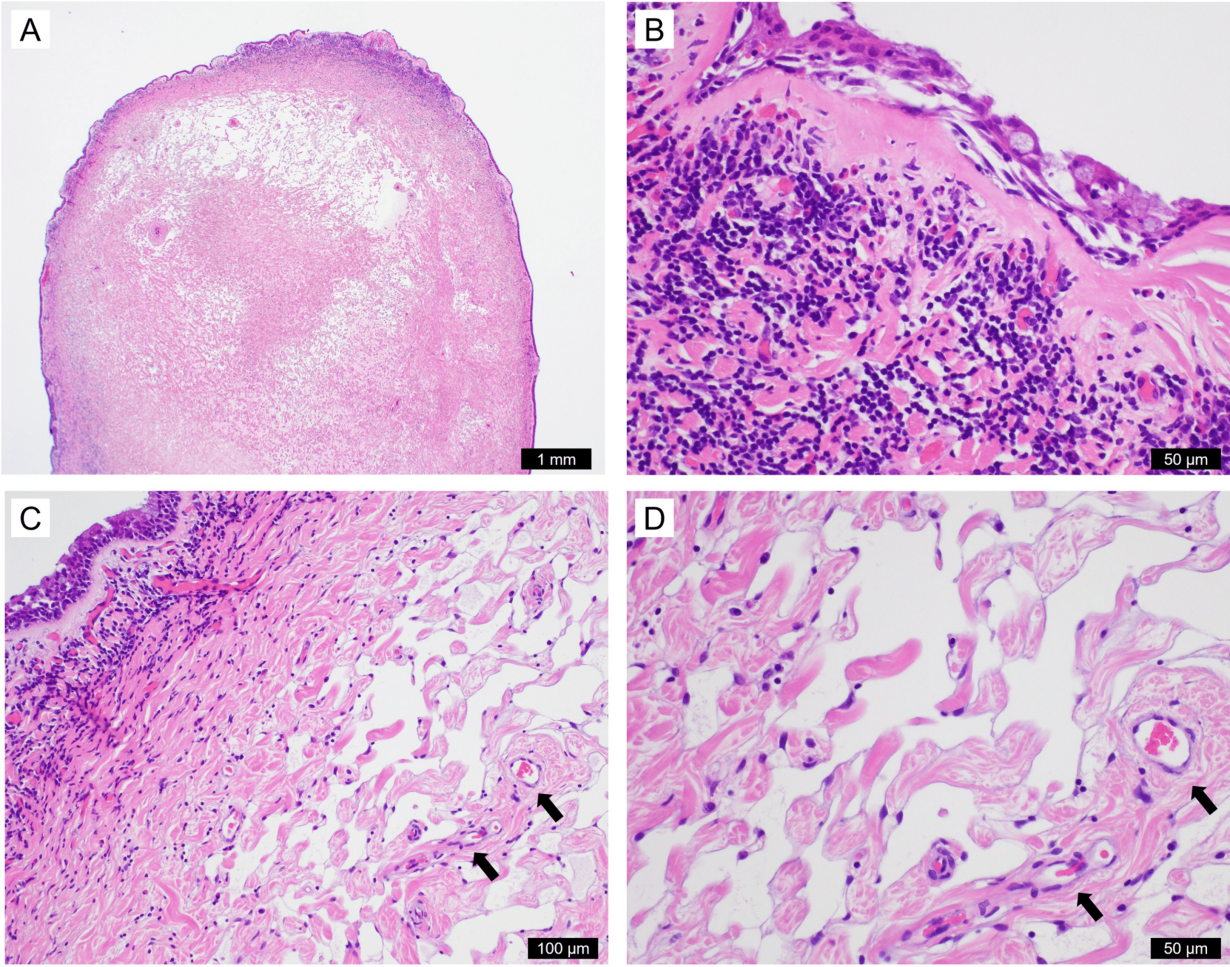
Microscopic findings of the inflammatory nasal polyp with benign lymphangioendothelioma/acquired progressive lymphangioma. Four photographs (A-D) of the nasal polyp are taken with hematoxylin and eosin-stained sections. The nasal polyp has a smooth surface lined by the respiratory epithelium (A, B) and abundant lymphatic vascular channels in the central portion (C, D). The clear stroma in A corresponds to the packed localization of dilated vascular channels. Lymphoplasmacytic infiltrate is observed beneath the epithelium with a thickened basement membrane (B). Vascular channels dissect the collagenous stroma, where existing vessels are indicated by arrows (C and D). The scale bars represent the figures.

Histologically, it appeared to be an inflammatory nasal polyp owing to the thickened basement membrane of the respiratory epithelium and abundant lymphoplasmacytic infiltrates beneath the epithelium (Figure 4). Eosinophils were scattered. Furthermore, diffused irregular vascular channels were observed in the central stromal portion of the polyp (Figure 4). These channels resembled lymphatic vessels; however, the infiltrative growth of these vessels was suggested because bundles of collagen and entrapped pre-existing vessels were observed inside the lesion (Figure 4).

**Figure 5:**
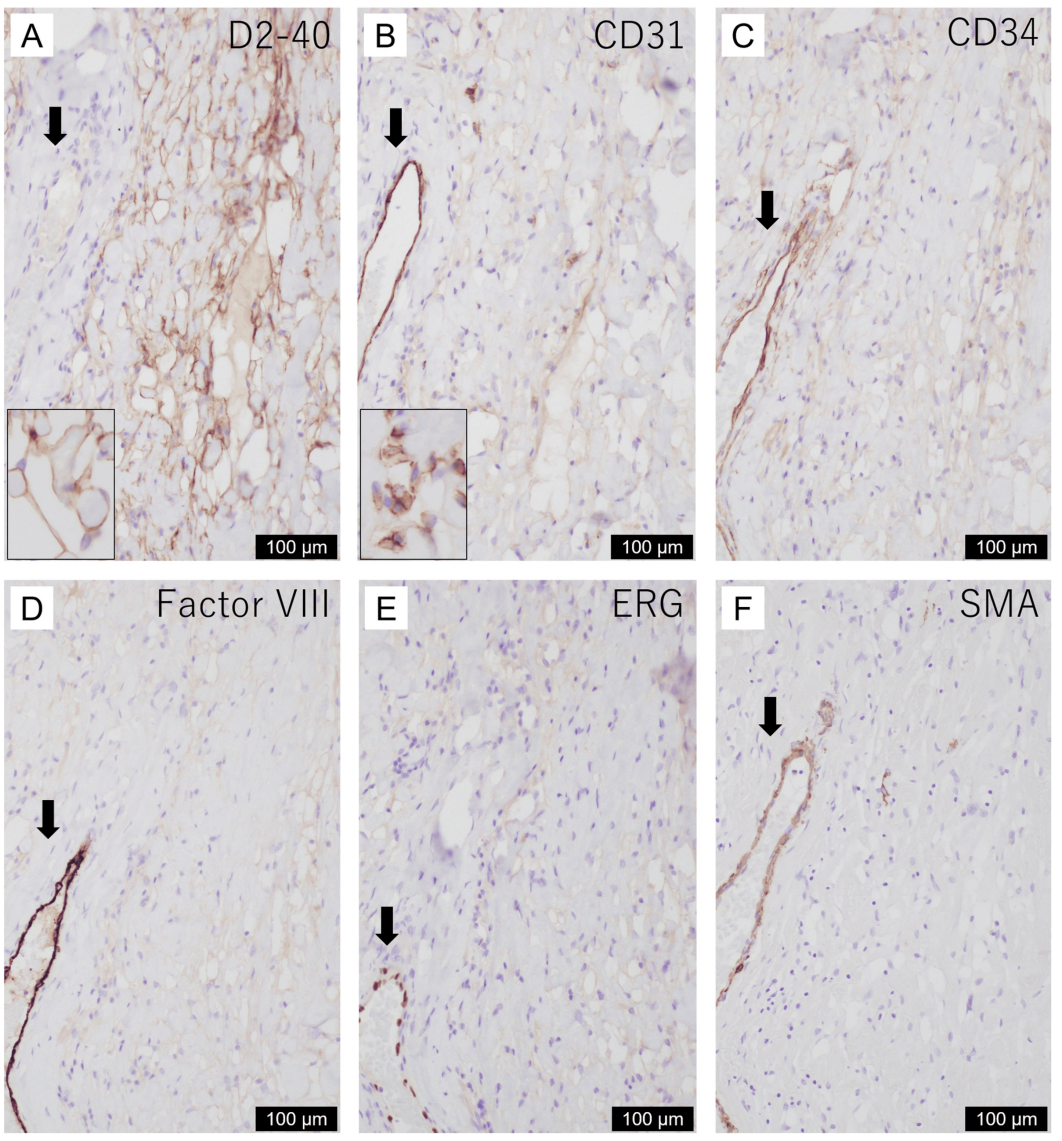
Immunohistochemical findings of the inflammatory nasal polyp with benign lymphangioendothelioma/acquired progressive lymphangioma. Endothelial cells of the dissecting vascular channels are largely positive for D2-40 (A), partly positive for CD31 (B), and negative for CD34 (C), factor VIII-related antigen (D), ERG (E), and alpha-smooth muscle actin (SMA) (F). Insets A and B show D2-40- and CD31-positive cells, respectively. The scale bars represent the figures.

Immunohistochemically (Figure 5), these channels were positive for D2-40, partly positive for CD31, and negative for epithelial membrane antigen, CD34, factor VIII-related antigen, E-26 transformation-specific (ETS)-related gene (ERG), Wilms tumor 1, estrogen receptor, progesterone receptor, androgen receptor, alpha-smooth muscle actin, desmin, and human herpesvirus 8 (HHV8). The endothelial cells were almost entirely negative for Ki67 (clone MIB1). Small vessels with CD34-positive endothelial cells and alpha-smooth muscle actin-positive pericytes were scattered inside the lesion, suggesting pre-existing vessels in the stroma (Figure 5).

Although the vascular channels were negative for some endothelial markers, such as CD34 and ERG, an inflammatory nasal polyp with BL/APL was considered based on histological findings and D2-40 and CD31 immunoreactivity.

The patient underwent successful surgical removal of the nasal polyp because the vascular channels were confined to the polyp and were not observed in the resection margin. A clinical follow-up of the right maxillary sinus lesions was performed. The patient provided informed consent for the publication of this report.

## Discussion

BL/APL is a rare vascular lesion with unclear etiology [3]. A recent literature review showed that BL/APL can present at any age, with a median age of 46 years. and no gender predilection [3]. BL/APL typically manifests as slow-growing erythematous patches or plaques, primarily on the extremities, followed by the breast, head, neck, and other areas such as the abdominal wall, chest, back, shoulder, buttock, axilla, and groin [3]. The nasal cavity is an unusual site for BL and APL. Our review of the PubMed database found two cases of lymphangioendothelioma in the nasal region [4,5]. However, these cases did not show benign lymphatic vascular proliferation with infiltrative channels dissecting collagen, which is a characteristic feature of BL/APLs. Therefore, we believe this is the first reported case of BL/APL in the nasal cavity.

In our case, the gross appearance of the nasal polyp was unusual for inflammatory nasal polyps, which are usually pink. The differential diagnosis of whitish nasal septum polyps includes lymphangiomatous polyps that histologically represent dilated vascular channels [6]. Lymphangiomatous polyps were histologically ruled out because of the presence of dissecting lymphatic channel growth. The white appearance of this polyp may be related to the abundance of stromal lymphatic fluid owing to BL/APL.

Angiofibromas, which usually arise in the nasopharynx or posterolateral nasal cavity wall, can also arise from the nasal septum [7]. Using contrast-enhanced computed tomography, angiofibroma can be clinically differentiated from BL/APL because they show prominent vascularity with marked contrast enhancement [7].

BL/APL can histologically mimic an aggressive vascular neoplasm, such as a well-differentiated angiosarcoma or Kaposi sarcoma, owing to its infiltrative growth pattern [1]. Angiosarcoma was ruled out in this tumor because of the absence of cellular atypia. Although HIV infection was not clinically tested, Kaposi sarcoma was ruled out because this tumor was immunohistochemically negative for HHV8, and BL/APL infection with HHV8 has not been previously reported [3]. Although pseudoangiomatous stromal hyperplasia observed in the breast was included in the differential diagnosis, this was unlikely because of the absence of CD34-positive stromal proliferation and hormone receptor expression. Meningeal hamartoma is also unlikely because of the lack of immunohistochemical expression of epithelial membrane antigen and progesterone receptor. Our case was histologically consistent with BL/APL because of the presence of infiltrating D2-40-positive lymphatic channels lined with plump endothelial cells. However, the immunohistochemical profile of the lesion was atypical. Previous studies on immunohistochemical profiling of BLE/APL included D2-40 (16/16), lymphatic vessel endothelial hyaluronan receptor 1 (LYVE1, 2/2), prospero homeobox protein 1 (PROX1, 5/5), CD31 (18/19), CD34 (16/16), factor VIII-related antigen (12/24), WT1 (2/8), and ERG (2/2) [1,8]. Previous studies on BL/APL have shown that all cases are positive for CD34, although one showed inconsistent results for CD34 [9]. Although we could not explain why the lesion was negative for CD34 and ERG, BL/APL was considered because of its typical histological features and D2-40 and CD31 immunoreactivity.

 Grossly, BL/APL typically forms flat lesions and sometimes papules or nodules, according to a literature review [3]. Considering the typical gross appearance of BL/APL, childhood asthma, and chronic mucosal inflammation, the pedunculated polypoid lesion in our case appeared to be mainly caused by an inflammatory nasal polyp rather than BL/APL. Therefore, we concluded that the lesion was an inflammatory nasal polyp containing the BL/APL. In the present case, the pathogenesis of BL/APL may have been related to inflammatory nasal polyps or inflammation. However, its pathogenesis is speculative, and future cases must be identified. No association between Kikuchi disease and BL/APL has been reported previously.

Two nodular lesions in the right maxillary sinus were identified on contrast-enhanced computed tomography and followed up. Although no cases of BL/APL derived from the maxillary sinuses have been reported, these two lesions may be BL/APL, as they can be seen multifocally [2].

## Conclusions

In conclusion, this case report describes a 17-year-old Japanese man with a rare inflammatory nasal polyp, including BL/APL. The successful surgical removal of the polyp enabled diagnosis. BL/APL is a benign condition that is usually observed on the skin, and this is the first reported case of BL/APL occurring in the nasal cavity in our review of the PubMed database. When a pedunculated white nasal polyp is observed, BL/APL can be included as a differential diagnosis. Whether this condition often forms polyps in the nasal cavity or whether tissue development is associated with inflammatory polyps needs to be verified by collecting more cases.
